# Assessing Plyometric Ability during Vertical Jumps Performed by Adults and Adolescents

**DOI:** 10.3390/sports6040132

**Published:** 2018-10-27

**Authors:** Brandon W. Snyder, Shawn N. Munford, Chris Connaboy, Hugh S. Lamont, Shala E. Davis, Gavin L. Moir

**Affiliations:** 1 Department of Exercise Science, East Stroudsburg University of Pennsylvania, East Stroudsburg, PA 18301, USA; bsnyder16@po-box.esu.edu (B.W.S.); smunford@po-box.esu.edu (S.N.M.); sdavis@esu.edu (S.E.D.); 2 Neuromuscular Research Laboratory, University of Pittsburgh, Pittsburgh, PA 15203, USA; connaboy@pitt.edu; 3 Department of Kinesiology, Coastal Carolina University, Conway, SC 29528, USA; hlamont@coatal.edu

**Keywords:** countermovement jump, drop jump, stretch-shortening cycle, reactive strength index

## Abstract

The purpose of this study was to compare different methods for assessing plyometric ability during countermovement (CMJ) and drop jumps (DJ) in a group of adults and adolescents. Ten resistance-trained adult men (age: 22.6 ± 1.6 years) and ten adolescent male basketball players (age: 16.5 ± 0.7 years) performed a CMJ and a DJ from a height of 0.40 m. Jump height (JH), contact time, normalized work (W_NORM_), and power output (PO_NORM_) during the absorption and propulsion phases were calculated from force platforms and 3-D motion analysis data. Plyometric ability was assessed using the modified reactive strength index (RSI_MOD_ during CMJ) and the reactive strength index (RSI during DJ) as well as three indices using propulsion time, propulsion work (PWI), and propulsion power. Adults jumped significantly higher than adolescents (mean difference [MD]: 0.05 m) while JH (MD: 0.05 m) and ground contact time (MD: 0.29 s) decreased significantly from CMJ to DJ. W_NORM_ (MD: 4.2 J/kg) and PO_NORM_ (MD: 24.2 W/kg) during the absorption phase of CMJ were significantly less than these variables during the propulsion phases of the jumps. The reactive strength index variants increased significantly from the CMJ to DJ (MD: 0.23) while all other plyometric indices decreased significantly. Neither RSI_MOD_ nor RSI contributed significantly to the prediction of JH during CMJ and DJ, respectively, while PWI was able to explain ≥68% of the variance in JH. Variants of the reactive strength index do not reflect the changes in mechanical variables during the ground contact phase of CMJ and DJ and may not provide an accurate assessment of plyometric ability during different vertical jumps.

## 1. Introduction

Plyometric training has been shown to be an effective means of improving measures of athletic ability including vertical jump, sprint running, and change of direction tasks [1,2]. Plyometric exercise such as countermovement jumps (CMJ) and drop jumps (DJ) incorporate the stretch-shortening cycle (SSC) whereby the musculotendinous units are initially lengthened prior to shortening up to takeoff [3]. This coupling of an initial active lengthening of the musculotendinous unit confers an increase in the mechanical output during the subsequent shortening phase as evidenced by greater power outputs recorded during CMJ that incorporate the SSC compared to those during a static jump where the SSC is absent [4,5,6]. The mechanisms underpinning the enhanced mechanical output during movements incorporating the SSC include strain potential energy contributions, reflex activation, and alterations in the crossbridge dynamics prior to the shortening phase [3] and the mechanics of the movement task have been proposed as influencing the relative contributions of these different mechanisms [7]. However, plyometric ability, defined as the ability to utilize the SSC during movement tasks, has been shown to be a trainable characteristic with improvements realized following a period of resistance training [8,9].

The reactive strength index (RSI), defined as the ratio between jump height and ground contact time, has been proposed as a means of assessing plyometric ability during jumping tasks [10]. The RSI was originally developed to assess plyometric ability during DJ [11] but has been modified for use in the assessment of plyometric ability in any vertical plyometric task. The modified RSI (RSI_MOD_) uses the time to takeoff (defined as the time from the initiation of the movement to takeoff during a jumping task) as the denominator of the ratio and therefore allows the assessment of plyometric ability during CMJ tasks [12]. From a mechanical perspective, the variants of RSI will increase in proportion to the square of the net impulse that an athlete is able to generate during the ground contact phase of a jumping task, decreasing in proportion with an increase in body mass and with an increase in ground contact time during the task [3]. Therefore, a high RSI or RSI_MOD_ would reflect the application of a large relative impulse in a short period of time. Such a mechanical characteristic would appear to be relevant to a variety of athletic tasks including jumping, sprint running, and change of direction and indeed the variants of RSI have been shown to differentiate between athletes from different sports [13] and identify different force-time and power-time characteristics during jumping tasks [14]. Furthermore, the RSI variants have been shown to be reliable [15,16,17,18]. These characteristics have resulted in the use of the RSI and RSI_MOD_ to select the intensity of plyometric exercises and monitor the adaptations to plyometric training methods [10,15,19,20].

Despite its widespread use, measures of RSI and RSI_MOD_ may not fully reflect the plyometric capabilities of athletes as neither accounts for the specific mechanical factors associated with the absorption and propulsion phases of ground contact during plyometric jumping exercises. Specifically, the absorption phase begins when the athlete first contacts the ground (during DJ) or when the center of mass begins to descend (the initiation of the countermovement during CMJ) and ends when the vertical motion of the center of mass has been arrested while the propulsion phase follows absorption and is defined as the phase between the first positive vertical motion of the center of mass until takeoff [3]. The absorption and propulsion phases of ground contact represent times when the active musculotendious units are lengthening (absorbing work) and shortening (generating work and/or returning energy), respectively, during the SSC and the plyometric ability is affected by the mechanical characteristics of these two phases. For example, greater enhancements in jump performance can be accrued when the magnitude and rate of loading during the absorption phase are increased, such as when performing a DJ compared to performing a CMJ [21,22]. However, information about the mechanical characteristics of the two phases (e.g., impulse of the ground reaction force, work, power output) are masked in measures of RSI and RSI_MOD_ and so these indices might not be sensitive to mechanical alterations during the execution of plyometric activities. Previous researchers have applied the RSI calculation in the separate absorption and propulsion phases of ground contact during DJ tasks in youth basketball players [23], but the new indices were limited to the duration of these phases only. An investigation into the use of other mechanical variables in the assessment of plyometric ability therefore appears warranted.

Variants of RSI are frequently used in the assessment of youth and adolescent athletes [24,25,26]. There is evidence that the developmental factors associated with aging may influence the plyometric abilities of the performer. Specifically, it has been demonstrated that the children have limited joint extension during the propulsion phase of jumping tasks compared to adults [27] while the active stiffness of the neuromuscular system increases as children grow older [28,29]. These mechanical changes may result in quantifiable changes in the plyometric ability across the lifespan. However, such changes in plyometric ability during jumping tasks may be masked with traditional measures such as RSI and RSI_MOD_ that are potentially insensitive to mechanical alterations during the execution of plyometric activities. Therefore, the purpose of the present study was to compare different methods for assessing plyometric ability during CMJ and DJ tasks performed by adults and adolescents.

## 2. Materials and Methods

### 2.1. Subjects

Ten resistance-trained men (age: 22.6 ± 1.6 years; height: 1.80 ± 0.10 m; mass: 85.7 ± 8.6 kg; 1-RM parallel back squat: 166.8 ± 24.6 kg) and ten adolescent male basketball players (age: 16.5 ± 0.7 years; height: 1.78 ± 0.07 m; mass: 69.5 ± 9.1 kg) volunteered to participate in this study which was approved by the Institutional Research Board of East Stroudsburg University. The adults reported having at least two years of exposure to regular resistance training (defined as a minimum of three resistance training sessions per week) and the adolescents had played competitively for their high school basketball team for at least one year. All subjects were free from musculoskeletal injuries in the six month period prior to data collection.

### 2.2. Procedures

Each subject completed two CMJ trials and two trials of a DJ from a height of 0.40 m. The drop height of 0.40 m has been used in both the assessment and training of plyometric abilities in youth and adolescent populations as well as adults [18,30,31,32]. An arm swing was restricted during all jumps by having the subjects place the hands on their hips and each subject completed two jumps under each condition with the mechanical data being averaged across the two jumps. The order of the jumps during each session was counterbalanced across the subjects. Prior to the jumps each subject participated in a warm-up comprising dynamic activities (e.g., bodyweight squats, lunges, high kicks, high knees, repeated jumps in-place) and the subjects were required to perform a practice of each jump type prior to data collection. The instruction of “jump as high and as fast as you can” was provided prior to the execution of each jump. During the DJ the participants were instructed to step out from the box one foot at a time rather than jumping from the box.

### 2.3. Collection of Mechanical Data

Force platforms (Kistler Type 9286AA, Amherst, NY, USA; 1000 Hz) were used to record the vertical ground reaction force (GRF) during each jump from which the descent (absorption phase) and ascent (propulsion phase) of the center of mass (CM) during ground contact was determined. The digital GRF signal was not subjected to filtering prior to processing. The vertical velocity of the CM at initial ground contact during the DJ task was determined from a synchronized 3-D motion analysis system (Vicon, Oxford, UK; 200 Hz). Specifically, the average vertical position of four retro-reflective markers placed around the pelvis of each subject (left and right anterior and posterior superior iliac spines), was differentiated with respect to time using the first central difference method to provide the vertical velocity of the CM as the subject descended from the box. It was assumed that the average location of the markers would reflect that of the CM given that the jumps were performed with the hands placed on the hips. The position data of the four retro-reflective markers was smoothed using a generalized cross-validated quantic spline procedure prior to differentiation. The ground contact phase of CMJ started when the vertical GRF fell > 10 N below bodyweight (representing the initiation of the countermovement) and ended at takeoff when the vertical GRF < 10 N. Bodyweight was calculated as the average vertical GRF across a 2 s period when the subject was stood stationary on the force platforms prior to the initiation of the countermovement during the CMJ. The ground contact phase of the DJ started when the vertical GRF > 10 N, with takeoff occurring when the GRF < 10 N. The following mechanical variables were then calculated using a custom program (Excel 2013, Microsoft Corporation, Redmond, WA, USA).

#### 2.3.1. Jump Height (JH)

The instantaneous vertical velocity of the CM, calculated as the time integral of the net vertical GRF using the trapezoid method, was identified at the point of takeoff. The following equation of motion was then used to calculate JH:

$$\mathrm{Jump}\;\mathrm{height}=\frac{v^{2}}{2g}$$

where *v* is the vertical velocity at takeoff, *g* is gravitational acceleration.

#### 2.3.2. Ground Contact Time (t_GC_)

The time from the start to the end of the ground contact phase represented ground contact time.

#### 2.3.3. Duration of Absorption Phase (t_Ab_)

The time from the beginning of the ground contact phase until the greatest negative displacement of the CM during ground contact represented t_Ab_.

#### 2.3.4. Duration of the Propulsion Phase (t_Pr_)

The time from the greatest negative displacement of the CM during ground contact until takeoff represented t_Pr_.

#### 2.3.5. Normalized Work (W_NORM_)

The work performed on the CM during the ground contact phase of each jump was calculated as the time integral of the instantaneous power output using the trapezoid method. Work was normalized to body mass and separated into that performed during the absorption phase (from the start of ground contact phase until the greatest negative displacement of the CM [W_AbNORM_]) and that performed during the propulsion phase (from the greatest negative displacement of the CM during ground contact until takeoff [W_PrNORM_]) for each jump. W_AbNORM_ was converted to a positive value for ease of comparison.

#### 2.3.6. Normalized Power Output (PO_NORM_)

The instantaneous power output was calculated as the product of the instantaneous vertical GRF and vertical velocity of the CM during ground contact. The vertical velocity of the CM was calculated as the time integral of the normalized net vertical GRF during the ground contact phase of each jump. The instantaneous power output was normalized to body mass and averaged across the absorption (PO_AbNORM_) and propulsion phases (PO_PrNORM_) of ground contact. PO_AbNORM_ was converted to a positive value for ease of comparison.

#### 2.3.7. Plyometric Indices

Five different plyometric indices were calculated from the mechanical data, as shown in Table 1.

The RSI_MOD_ and RSI were calculated following the recommendations of Ebben and Petushek [12] where RSI_MOD_ is the ratio of jump height to ground contact time (referred to as time to takeoff by others [13]) during the CMJ task while RSI is the ratio of jump height during the DJ task to the duration of the ground contact phase. Both RSI_MOD_ and RSI therefore increase in proportion to jump height but are inversely proportional to ground contact time. The three other plyometric indices were developed to retain the proportionality with jump height by maintaining this as the numerator but each used a different mechanical variable as the denominator. However, all indices maintained the inverse proportionality between the calculated value and the denominator, in keeping with the RSI_MOD_ and the RSI. The propulsion time index (PTI) was calculated with ground contact time replaced by the ratio of propulsion time to absorption time as the denominator such that the index could be increased through the completion of a shorter propulsion phase. The denominator used in the propulsion work index (PWI) was the inverse of the ratio of the normalized work completed during the propulsive phase to that completed during the absorption phase. This index could therefore be increased through the completion of a greater amount of work during the propulsive phase (the denominator is the inverse of the propulsion-absorption work ratio) and so the PWI reflects the loss of work between the absorption and propulsive phases of ground contact. Finally, the propulsion power output index (PPI) used the inverse of the ratio of the normalized power output during the propulsive phase to that during the absorption as the denominator. The PPI therefore reflects the loss of power output between the absorption and propulsion phases during ground contact of the jumping task.

### 2.4. Statistical Analysis

All statistical analyses were performed using the Statistical Package for the Social Sciences (SPSS version 20.0. Chicago, IL, USA). Measures of central tendency and spread of data were represented as means and standard deviations (±SD). A two-way analysis of variance (ANOVA) was used to determine the differences in the variables of JH, ground contact time, and the plyometric indices with repeated measures on one factor (jump condition [two levels]), and age-group (two levels) entered as a between-subject factor. A three-way ANOVA was used for the mechanical variables (duration of phases, normalized work, and power output) during the absorption and propulsion phases with repeated measures on two factors (jump condition [two levels]; jump phase [two levels]) and age-group (two levels) entered as a between-subject factor. Pairwise comparisons with Bonferroni corrections were used to establish where any significant differences were located.

A multiple linear regression analysis was performed to determine the plyometric indices that best predicted JH. If the ANOVA model revealed statistically significant differences between the adults and the adolescents on the five different plyometric indices then separate regression models were constructed for each age-group; if there were no statistically significant age-group differences then the data for the adolescent and adult subjects was pooled. The predictors (RSI_MOD_, RSI, PTI, PWI, and PPI) were entered in a stepwise manner. The level of statistical significance for all analyses was set at *p* ≤ 0.05.

## 3. Results

The mechanical variables during the CMJ and DJ are displayed in Table 2.

JH was significantly greater during CMJ compared to the DJ condition (mean differences: 0.05 m; *p* < 0.05) and the adults jumped significantly higher than the adolescents (mean difference: 0.05 m; *p* = 0.04). Ground contact time was significantly greater during CMJ compared to DJ (mean difference: 0.29 s; *p* < 0.05) and the adolescent group produced significantly shorter ground contact times across the two jumping conditions compared to the adults (mean difference: 0.16 s; *p* < 0.05). The duration of the propulsion phase was significantly shorter than that of the absorption phase across both jumping tasks (mean difference: 0.10 s; *p* < 0.05) and a significant interaction between the jump condition and the ground contact phase revealed that the effect was caused by a significantly longer absorption phase associated with the CMJ condition (mean difference: 0.25 s; *p* < 0.05).

A greater amount of work was performed during the ground contact of the DJ compared to the CMJ (mean difference: 1.5 J/kg; *p* < 0.05) and the adults performed greater amounts of work across the two jump conditions compared to the adolescents (mean difference: 1.6 J/kg; *p* < 0.05). The work performed during the absorption phase of the CMJ was significantly less than that completed during the propulsion phase of the CMJ and both the absorption and propulsion phases of the DJ (mean difference: 4.2 J/kg; *p* < 0.05). Power output was significantly greater during the DJ compared to the CMJ (mean difference: 12.9 W/kg; *p* < 0.05). The power output during the absorption phase of the CMJ was significantly less than that completed during the propulsion phase of the CMJ and both the absorption and propulsion phases of the DJ (mean difference: 24.2 W/kg; *p* < 0.05).

Table 3 shows the five different plyometric indices calculated for each of the jumping conditions.

The adolescent group produced greater RSI_MOD_ and RSI values compared to the adults, although the difference was not statistically significant (mean difference: 0.11, *p* = 0.063). However, the RSI during the DJ task was significantly greater than the RSI_MOD_ recorded during the CMJ task for both groups (mean differences: 0.23, *p* < 0.001). The increase from RSI_MOD_ to RSI by the adolescents was greater than that by the adults although the difference was not statistically significant (mean difference: 0.11, *p* = 0.057). In contrast, all of the other plyometric indices decreased significantly from the CMJ to DJ (PTI mean difference: 0.40; PWI mean difference: 0.60; PPI mean difference: 1.43; *p* < 0.05).

Because the ANOVA model revealed that the differences in the plyometric indices between the two age-groups were not statistically significant the data from the adults and adolescents was pooled in the subsequent multiple linear regression analysis to determine the plyometric indices that best predicted JH. The statistics for the regression models to predict JH during the CMJ and JH during the DJ are shown in Table 4 and Table 5, respectively.

The PWI was a significant contributor to the prediction of JH during both the CMJ and the DJ, explaining 68% and 89% of the variance in JH during CMJ and DJ, respectively. Neither RSI_MOD_ nor RSI contributed significantly to the prediction of CMJ JH or DJ JH, respectively, and were excluded from the regression models.

## 4. Discussion

The purpose of the present study was to compare different methods for assessing plyometric ability during CMJ and DJ in adults and adolescents. It was found that ground contact times decreased significantly from the CMJ to the DJ task in both age groups as a result of significant reductions in the duration of both the absorption and propulsion phases. However, there was also a concomitant decrease in jump height by both age groups between the CMJ and the DJ. It has been shown that performance during jumping tasks incorporating the SSC is enhanced when the magnitude and rate of loading during the absorption phase are increased [21,22]. Indeed, Moran and Wallace [33] have noted that jump height increases in well-trained individuals when they perform DJ compared to CMJ as a result of the increased eccentric loading and therefore the lack of enhancement in jump height demonstrated in the present study may reflect the training status of the subjects used. Other researchers have noted that the technique used during a DJ task can affect jump height with the execution of a DJ with very brief ground contact times not necessarily resulting in greater jump heights [23,34]. This likely reflects the ability of the performer to utilize the SSC, that is, their plyometric ability. Therefore, the instruction to “jump as high and as fast as you can” as used in the present study may have constrained the technique of the participants during the jumping tasks preventing them from utilizing the SSC as effectively during the DJ compared to the CMJ.

In contrast to the changes in jump height, the normalized work and power output increased significantly during the absorption phase when the subjects performed the DJ compared the same variables during the absorption phase of the CMJ, reflecting the increased loading during the DJ compared to the CMJ. However, there were no significant differences reported for the work performed and the power output generated during the propulsion phases of the two jumps indicating that both groups of subjects were unable to utilize the SSC as effectively during the DJ as they were during the CMJ. Indeed, both W_NORM_ and PO_NORM_ decreased slightly from the absorption to the propulsion phase of the DJ for both groups of subjects, although the differences were not statistically significant. However, despite the alterations in W_NORM_ and PO_NORM_ during the absorption and propulsive phases indicating that the subjects’ plyometric ability was reduced when they performed the DJ, RSI_MOD_ during the CMJ was lower than the RSI attained during the DJ task. In contrast, all of the other plyometric indices decreased from CMJ to DJ. Furthermore, neither RSI_MOD_ nor RSI contributed significantly to the prediction of JH during the CMJ or DJ tasks. These findings may bring into question the use of the variants of the reactive strength index to select the intensity of plyometric exercises and monitor the adaptations to plyometric training methods [10,15,19,20]. Struzik et al. [23] calculated plyometric indices from the ratio of JH to the duration of the separate absorption and propulsion phases of ground contact during DJ tasks from different drop heights performed with different jumping techniques. The authors reported that the changes in these newly calculated indices closely matched the changes recorded in RSI, meaning that the new indices offered no further information on the plyometric ability of the participants. The PTI used in the present study used the ratio of propulsion time to absorption time in the calculation such that the index would be sensitive to the completion of a shorter propulsion phase relative to the absorption phase and this index responded differently to the RSI variants as a result of the increased loading associated with the DJ. PTI was also a significant contributor to JH during both CMJ and DJ tasks. The PWI and the PPI used the ratio of kinetic variables during the propulsion phase to those during the absorption phase such that these indices would be expected to increase in response to a greater amount of work performed (PWI) or a greater power output (PPI) during the propulsion phase relative to the absorption phase. Both PWI and PPI decreased significantly for both groups of subjects as they performed the DJ compared to the CMJ. Furthermore, the PWI was a significant contributor to the prediction of JH during both the CMJ and the DJ, explaining 68% and 89% of the variance in JH during CMJ and DJ, respectively. The PWI reflects the loss of work between the absorption and propulsive phases of ground contact during jumping tasks and the coefficients for this plyometric index when it was the only predictor entered into the regression models revealed that an increase of one unit in PWI would result in an increased JH of 0.20 and 0.65 m for the CMJ and DJ tasks, respectively. Therefore, practitioners should consider using the PWI to assess the plyometric ability of their athletes.

It has been noted that there are biomechanical changes evident during jumping tasks as children grow and develop across the lifespan [27,28,29] and that these changes are likely to influence the plyometric abilities, possibly resulting in quantifiable differences between adults and adolescents. It was found in the present study that the adolescent performers produced significantly shorter ground contact times across the two jumping conditions compared to the adults. This difference may reflect greater stiffness in the adolescent subjects used here compared to the adults, although such a finding would tend to run counter to previous research [28,29]. It is possible that the shorter ground contact times also reflects a limited joint extension during the propulsion phase of jumping that has been observed in children performing jumping tasks [27]. However, neither the duration of the absorption nor propulsion phases were significantly shorter in the adolescent subjects compared to the adults with age-group differences only being statistically significant when the two phases were combined. It was found that the adults in the present study performed significantly greater amounts of normalized work across the two jump conditions compared to the adolescents. This was caused by the combination of greater work during both the absorption and propulsion phases of the jumping tasks and likely reflects their greater muscular strength. Interestingly, none of these mechanical differences were reflected in the newly developed plyometric indices (PTI, PWI, PPI) with the age-group differences being non-significant. The differences in ground contact times between the two groups was reflected somewhat in the RSI_MOD_ and RSI values which were greater in the adolescents compared to the adults, although the differences were not statistically significant. The lack of statistically significant difference in the RSI_MOD_ and RSI values was the result of the significantly greater heights attained by the adults in both jumping tasks despite their longer ground contact times. It may be concluded that the plyometric indices assessed here were not sensitive enough to detect the mechanical differences between the two subject groups used or that the sample size was simply too small to detect any statistical differences. However, it may also be concluded that the two group did not actually differ in their plyometric abilities, reflecting the training status of the subjects used in the present study. Specifically, the adults were resistance-trained but did not have extensive experience in plyometric training methods. Although the jump heights attained by the adults during both the CMJ and DJ in the present study were comparable to those of samples used in previous research performing the same jumping tasks [13,14,18], the ground contact times were much greater than those reported for elite basketball players [18]. This implies that the adults used in the present study required a substantially greater ground contact phase to attain their jump heights compared to those athletes engaged in extensive jumping activities in their sport, highlighting a potential shortcoming in the plyometric ability of the adults used in the present study. The adolescents used in the present study were competitive basketball players although they had not been exposed to regular plyometric training. They attained jump heights in the CMJ that were comparable to those reported in a cohort of elite adolescent basketball players aged between 14 and 15 years [32]. However, the performance of the adolescents in the present study during the DJ was below that of the elite adolescent basketball players used by Santos and Janeira [32]. It is possible that both the adults and the adolescents used in the present study might have possessed similar plyometric abilities despite any developmental or strength differences. Therefore, future research should perform assessments on the newly-developed plyometric indices using large samples of adults and adolescents that have an extensive background in plyometric activities and training. Furthermore, the indices should be investigated using a variety of different plyometric jumping tasks. In this regard, Schmidtbleicher [35] noted that jumping tasks that are performed with ground contact times >0.25 s are considered slow SSC activities and so the data presented here do not relate to fast SSC activities.

The reliability of the newly-developed plyometric indices should be assessed before practitioners and researchers use them. Reliability refers to the reproducibility of a specific measure and is an important consideration when determining individual responses to a training regimen and also in the selection of appropriate sample sizes for experiments [36]. Variants of RSI have been shown to be reliable [15,16,17,18] and such assessments are still required with PTI, PWI, and PPI. Furthermore, the validity of the newly developed plyometric indices presented here requires investigation. The validity of a measure refers to the degree to which the test measures what it is supposed to measure [37]. The data presented here would bring the validity of RSI_MOD_ and RSI as measures of plyometric ability into question. However, RSI_MOD_ has been shown to differentiate between athletes from different sports [13], thereby displaying discriminative validity, yet the sensitivity of the index to training-induced adaptations has not always been demonstrated [38,39]. Previous researchers have highlighted the need to determine the relationship between changes in assessments of muscular strength and changes in performance variables following the training regimens [40,41]. It is therefore recommended that training studies be performed to investigate the adaptations to resistance training and plyometric training methods in order to determine the sensitivity of the newly-developed plyometric indices (PTI, PWI, PPI) to training adaptations.

## 5. Conclusions

RSI_MOD_ and RSI do not reflect the changes in mechanical variables during the ground contact phase of CMJ and DJ and may not therefore provide an accurate assessment of plyometric ability during different jumping tasks. Practitioners and researchers should consider using the PWI to assess the plyometric ability of their athletes.

## Figures and Tables

**Table 1:** The calculations for the five different plyometric indices *.

Plyometric Index	Calculation
Modified reactive strength index (RSI_MOD_)	$\frac{\mathrm{CMJ}\;\mathrm{JH}}{t_{\mathrm{GC}}}$
Reactive strength index (RSI)	$\frac{\mathrm{DJ}\;\mathrm{JH}}{t_{\mathrm{GC}}}$
Propulsion time index (PTI)	$\frac{\mathrm{JH}}{\left(t_{\mathrm{pr}}÷t_{\mathrm{Ab}}\right)}$
Propulsion work index (PWI)	$\frac{\mathrm{JH}}{{\left(W_{\mathrm{PrNORM}}÷W_{\mathrm{AbNORM}}\right)}^{-1}}$
Propulsion power index (PPI)	$\frac{\mathrm{JH}}{{\left({\mathrm{PO}}_{\mathrm{PrNORM}}÷{\mathrm{PO}}_{\mathrm{AbNORM}}\right)}^{-1}}$

* CMJ JH is jump height during the countermovement jump task; t_GC_ is ground contact time; DJ JH is jump height during the drop jump task; JH is jump height; t_Pr_ is the duration of the propulsion phase; t_Ab_ is the duration of the absorption phase; W_PrNORM_ is the normalized work performed during the propulsive phase; W_AbNORM_ is the normalized work performed during the absorption phase; PO_PrNORM_ is the average normalized power output during the propulsive phase; PO_AbNORM_ is the average power output during the ground absorption phase.

**Table 2:** The mechanical variables during the countermovement jump and the drop jump performed by the adults and the adolescents. Values are means ± standard deviation *.

Mechanical Variable	Adults		Adolescents	
	CMJ	DJ_40_	CMJ	DJ_40_
Jump height (m)	0.40 ± 0.06	0.36 ± 0.07	0.36 ± 0.05	0.30 ± 0.04
Ground contact time (s)	0.83 ± 0.08	0.57 ± 0.07	0.71 ± 0.09	0.38 ± 0.09
**Mechanical variables during the absorption phase**				
Duration (s)	0.53 ± 0.06	0.28 ± 0.04	0.46 ± 0.06	0.18 ± 0.05
Normalized work (J/kg)	4.0 ± 0.4	8.9 ± 0.7	3.1 ± 0.6	6.6 ± 0.7
Normalized power (W/kg)	7.7 ± 1.2	31.5 ± 4.8	6.2 ± 1.1	33.9 ± 6.4
**Mechanical variables during the propulsion phase**				
Duration (s)	0.31 ± 0.03	0.29 ± 0.03	0.24 ± 0.04	0.20 ± 0.04
Normalized work (J/kg)	8.9 ± 0.8	8.3 ± 0.9	7.9 ± 0.8	6.2 ± 0.7
Normalized power (W/kg)	29.2 ± 3.4	28.3 ± 4.1	31.6 ± 4.7	32.5 ± 4.4

* CMJ is countermovement vertical jump; DJ_40_ is drop jump from a 0.40 m drop height.

**Table 3:** The different indices of plyometric ability during the countermovement jump and the drop jump performed by the adults and the adolescents. Values are means ± standard deviations *.

Plyometric	Adults		Adolescents	
Index	CMJ	DJ_40_	CMJ	DJ_40_
RSI_MOD_	0.48 ± 0.09	-	0.52 ± 0.11	-
RSI	-	0.64 ± 0.16	-	0.82 ± 0.20
PTI	0.69 ± 0.16	0.34 ± 0.08	0.71 ± 0.17	0.27 ± 0.06
PWI	0.89 ± 0.24	0.34 ± 0.10	0.95 ± 0.22	0.29 ± 0.07
PPI	1.58 ± 0.54	0.33 ± 0.10	1.92 ± 0.30	0.55 ± 0.08

* CMJ is countermovement vertical jump; DJ_40_ is drop jump from a 0.40 m drop height; RSI_MOD_ is reactive strength index-modified; RSI is reactive strength index; PTI is propulsion time index; PWI is propulsion work index; PPI is propulsion power index.

**Table 4:** The multiple linear regression models to predict jump height during the countermovement jump from the different plyometric indices *.

Model	β Constant	*R* ^2^	Plyometric Index	β Coefficient	t-Statistic	*p*-Value
1	0.197	0.680	PWI	0.198	6.181	<0.001
2	0.171	0.818	PWI	0.390	6.603	<0.001
			PPI	−0.086	−3.589	0.002
3	0.138	0.883	PWI	0.337	6.487	<0.001
			PPI	−0.110	−5.148	<0.001
			PTI	0.176	2.991	0.009

* PWI is propulsion work index; PPI is propulsion power index; PTI is propulsion time index.

**Table 5:** The multiple linear regression models to predict jump height during the drop jump from the different plyometric indices *.

Model	β Constant	*R* ^2^	Plyometric Index	β Coefficient	t-Statistic	*p*-Value
1	0.127	0.885	PWI	0.645	11.792	<0.001
2	0.098	0.936	PWI	0.427	5.865	<0.001
			PTI	0.319	3.672	0.002
3	0.084	0.980	PWI	0.683	11.486	<0.001
			PTI	0.583	8.808	<0.001
			PPI	−0.469	−6.018	<0.001

* PWI is propulsion work index; PTI is propulsion time index; PPI is propulsion power index.
